# Prognostic plasma exosomal microRNA biomarkers in patients with substance use disorders presenting comorbid with anxiety and depression

**DOI:** 10.1038/s41598-021-84501-5

**Published:** 2021-03-18

**Authors:** Fengrong Chen, Lei Zou, Yicong Dai, Jiaxue Sun, Cheng Chen, Yongjin Zhang, Qingyan Peng, Zunyue Zhang, Zhenrong Xie, Hongjin Wu, Weiwei Tian, Xu Yu, Juehua Yu, Kunhua Wang

**Affiliations:** 1 NHC Key Laboratory of Drug Addiction Medicine, First Affiliated Hospital of Kunming Medical University, Kunming, 650032 Yunnan China; 2 Centre for Experimental Studies and Research, First Affiliated Hospital of Kunming Medical University, Kunming, 650032 Yunnan China; 3 Yunnan Institute of Digestive Disease, First Affiliated Hospital of Kunming Medical University, Kunming, 650032 Yunnan China; 4 Department of Organ Transplant, First Affiliated Hospital of Kunming Medical University, Kunming, 650032 Yunnan China

**Keywords:** Biomarkers, Psychiatric disorders

## Abstract

Psychiatric disorders such as anxiety and depression precipitated by substance use occurred during both use and withdrawal. Exosomes play significant roles in biological functions and regulate numerous physiological and pathological processes in various diseases, in particular substance use disorders (SUDs) and other psychiatric disorders. To better understand the role of exosomal miRNAs in the pathology of symptoms of anxiety and depression in patients with SUDs, we first isolated circulating exosomes from heroin-dependent patients (HDPs) and methamphetamine-dependent patients (MDPs) and identified exosomal miRNAs that were differentially expressed between patients and healthy controls (HCs). Furthermore, the correlations between exosomal DE-miRNAs and symptoms of anxiety and depression which were measured using Hamilton-Anxiety (HAM-A)/Hamilton-Depression (HAM-D) Rating Scales in the participants. Notably, the expression level of exosomal hsa-miR-16-5p, hsa-miR-129-5p, hsa-miR-363-3p, and hsa-miR-92a-3p showed significantly negative correlations with HAM-A scores in both HDPs and MDPs. But all of the 4 DE-miRNAs lost significant correlations with HAM-D scores in HDPs. Functional annotation analyses showed that the target genes of the DE-miRNAs were mainly enriched for “synapse”, “cell adhesion”, “focal adhesion” and “MHC class II protein complex”. Our study suggests that a set of circulating exosomal miRNAs were associated with anxiety and depression in SUD patients and may have clinical utility as diagnostic and prognostic biomarkers.

## Introduction

At least 35 million people suffer from substance use disorders (SUDs) globally^1^, which has led to major social and health concerns. As of December 2018, there were 2.4 million substance-dependent patients in China, with methamphetamine (MA)-dependent patients (MDPs) accounting for 56.1% of the total, and heroin-dependent patients (HDPs) for 37%^2^. Yunnan, which borders the notorious “Golden Triangle”, recorded 188,000 registered substance abusers in 2018^3^.

Substance abuse results in adverse health issues, including cardiovascular problems such as cerebrovascular events, strokes^4^, cardiomyopathy^5^, suppression of both innate and adaptive immunity^6^, and a high prevalence of infectious diseases such as human immunodeficiency virus (HIV) and hepatitis-C infection^6,7^. Chronic substance use is associated with impaired cognitive function, including deficits in attention, learning, and memory^8,9^. Notably, emerging evidence has suggested that depression and anxiety are common events in MDPs and HDPs, during both use^10,11^ and withdrawal^11,12^. A recent study revealed that 37.1% of patients with MA use disorder presented with MA-induced psychosis^13^, while another study reported that 51% of opioid-dependent individuals exhibited high rates of psychiatric disorders (30% of participants screened positive for moderate to severe depression and 21% for bipolar disorder)^14^. Nevertheless, there are difficulties in the diagnosis and treatment of psychiatric disorders precipitated by substance use.

Biomarkers are critical for the development of medical diagnostics and classification, therapeutics, and their respective uses in patient care^15^. Recently, significant research efforts have been devoted to depression and anxiety through the use of neuroimaging, genetics, epigenetics, and blood-based approaches in an attempt to identify pathogenic- and treatment-related biomarkers^16^. Changes in DNA^17^, epigenetic modifications (including DNA methylation and histone modifications)^18,19^, and miRNA level^20^ have all been used as biomarkers in neurological and psychiatric disorders. Additionally, neurotrophic factors such as brain-derived neurotrophic factor (BDNF), glial cell line-derived neurotrophic factor (GDNF), insulin-like growth factor-1 (IGF-1), and vascular endothelial growth factor (VEGF) have been investigated as potential biomarkers in psychiatric disorders, including SUDs^21–24^. Urine-based metabolites involved in metabolic pathways were deemed as a hopeful diagnostic method for patients with depression and anxiety disorders^25^. Nevertheless, existing biomarkers in cerebrospinal fluid (CSF) [e.g., monoamine and cocaine- and amphetamine-regulated transcript (CART) peptide identify biomarkers for psychiatric diagnosis]^26^ or positron emission tomography (PET) imaging [targeting serotonin transporter (5-HTT), 5-HTT and 5-HT1A, tau, neurofilament light, and neurogranin]^27–29^ are invasive or expensive, far from comprehensive, and often lack sensitivity and specificity.

Exosomes are extracellular vesicles (EVs) containing selectively packaged proteins, miRNAs, mRNAs, lncRNAs, and DNA and play significant roles in biological functions, including the transfer of biomolecules and regulation of numerous physiological and pathological processes in various diseases. In addition to cellular miRNAs, circulating miRNAs (including exosomal miRNAs) in plasma are deemed as potential biomarkers for disease diagnosis and prognosis^30^, which can be detected by PCR. Studies have shown that alterations in circulating miRNA profiles are strongly associated with disease progression as well as the regulation of immune activation and inflammation^31^, consequently, they can be used as biomarkers for cancer and infectious diseases^32,33^. For example, the level of plasma exosomal miRNAs that have been identified from HIV infection and/or heroin abuse can be used as biomarkers in immune regulation and neuroinflammation^34^. Furthermore, miRNAs and exosomes have been proposed as new diagnostic biomarkers for patients with anxiety^35^ and depression^36^. Owing to their stability and accessibility, exosomal biomarkers could provide greater sensitivity and specificity compared with biomarkers identified in conventional specimens such as serum or urine.

MiRNAs are small, noncoding RNAs that play an essential role in gene silencing and translational repression by binding to target mRNAs, thereby regulating gene expression under different physiological and pathophysiological conditions^37^. Recent studies have suggested that miRNAs played pivotal roles in the development of depression and anxiety, and have emerged as potential therapeutic targets and tools for use in the diagnosis and treatment responses of patients with depression and anxiety^38^. However, relatively few studies have focused on the role of circulating exosomal miRNAs in SUD patients^34^, while other two researches were performed on rats^39,40^. None of them were conducted in SUD patients with anxiety and depression.

In this study, we aimed to profile exosomal miRNAs to identify a set of exosomal miRNAs showing differential expression between HCs and SUD patients with symptoms of anxiety and depression. As shown in Fig. S1, we first isolated circulating exosomes from individuals with SUDs, and identified exosomal miRNAs that were differentially expressed between SUD patients and HCs. The identified DE-miRNAs were subsequently validated by quantitative reverse transcription PCR (RT-qPCR). Then, we established associations between exosomal DE-miRNAs and depression and anxiety scores in SUD patients, and evaluated the potential underlying biological functions and pathways associated with the key miRNAs in SUD patients with anxiety and depression by Gene Ontology (GO) and Kyoto Encyclopedia of Genes and Genomes (KEGG) analyses. These findings will contribute to identify potential exosomal miRNA biomarkers for the prediction of SUD-associated anxiety and depression.

## Results

### Demographics and hematological parameters of the study participants

The demographics of the study participants are displayed in Table 1, the age, gender and years of education of the MDPs and HDPs were matched to those of the HCs. A total of 20 hematological parameters were examined (Supplementary Table S1). Compared with the HCs, four of these parameters in both HDPs and MDPs, related to white blood cells, showed significantly increased values: White blood cell count (WBC), neutrophile (NEUT) count, basophil count (BASO#) and basophil percentage (BASO%) (all *p* < 5e−02). Two of the red blood cell-related parameters were also significantly altered: hematocrit (HCT) was increased in both MDPs and HDPs (all *p* < 5e−02), while the mean corpuscular hemoglobin concentration (MCHC) was decreased in both MDPs and HDPs (both *p* < 5e−02). Blood biochemistry parameters were also obtained for all study participants (Supplementary Table S1). Compared with that in HCs, the total protein (TP), Prealbumin (PAB), Albumin (ALB), globulin (GLB), and complement 3 (C3) concentration were increased in both MDPs and HDPs (all *p* < 5e−02). Whereas the ALB/GLB ratio was decreased in both groups of patients (both *p* > 5e−02). Liver function parameters were within the normal range; nevertheless, the aspartate aminotransferase (AST)/alanine aminotransferase (ALT) ratio was decreased in both MDPs and HDPs when compared with that of HCs (both *p* < 5e−02). Sodium (Na), magnesium (Mg), and phosphorus (P) concentrations in MDPs and HDPs were higher (all *p* < 5e−02) than those of HCs. No significant differences were detected in any of the other parameters among the three groups.

**Table 1:** Demographics characterisristics of study participants.

Parameters	HCs (M ± SD, n = 10)	HDPs (M ± SD, n = 10)	MDPs (M ± SD, n = 10)
Age (years)	36.71 ± 2.16	37.02 ± 2.29	36.93 ± 2.11
Education (years)	6.85 ± 1.61	6.80 ± 1.06	6.75 ± 1.4
Married (%)	90	50	30
BMI	22.79 ± 3.20	21.49 ± 3.42	20.90 ± 2.38
Smoking status	10/10	10/10	10/10
Drug	–	Heroin only	Methamphetamine only
Numbers of relapse (times)	–	2.0 ± 0.31	2.0 ± 0.27
Total duration of drug use (years)	–	7.3 ± 1.06	7.9 ± 0.76

*M* mean, *SD* standard deviation, *MDPs* methamphetamine-dependent patients, *HDPs* heroin-dependent patients, *HCs* healthy controls, *BMI* Body Mass Index.

### Behavioral characteristics of the study participants

Data for all three behavioral scales aforementioned were collected for all the study participants (Table 2). The degree of drug craving was assessed by VAS and the scores among the three age-, gender-, and education-matched groups are shown in Table 2. Patients with SUDs were associated with higher VAS scores (MDPs: *p* = 2.19e−03; HDPs: *p* = 5.5e−03) compared with HCs. No significant differences were found between MDPs and HPDs (*p* > 5e−03).

**Table 2:** Behavioral characteristics of study participants.

	HCs (M ± SD, n = 10)	HDPs (M ± SD, n = 10)	Adjusted *p* value (HC vs MDPs)	MDPs (M ± SD, n = 10)	Adjusted *p* value (HC vs MDPs)
VAS craving scale score	0	2.7 ± 1.95	**5.5e–03**	3.0 ± 2.36	**2.1e−03**
HAM-A	4.9 ± 2.42	15.3 ± 5.76	** < 1.0e−04**	13.6 ± 3.86	**3.0e−04**
HAM-D	8.2 ± 3.82	15.2 ± 5.33	**8.8e−03**	11.7 ± 5.23	0.26

*M* mean, *SD* standard deviation, *VAS* Visual-Analogue Craving Scale, *MDPs* methamphetamine-dependent patients, *HDPs* heroin-dependent patients, *HCs* healthy controls.

As shown in Table 2, the intergroup analysis of the HAM-A scale revealed that both MDPs and HDPs presented significantly higher scores than the HCs (both *p* < 1e−02). A comparison of the median scores of the HAM-D scale among the groups indicated that significant differences existed between the HCs and HDPs (*p* = 8.89e−03), but not between the HCs and HDPs (*p* > 5e−02). No differences in HAM-A or HAM-D scale scores were observed between the MDPs and HDPs (*p* > 5e−02).

### Characteristics of exosomes obtained from the plasma of the study participants

**Figure 1:**
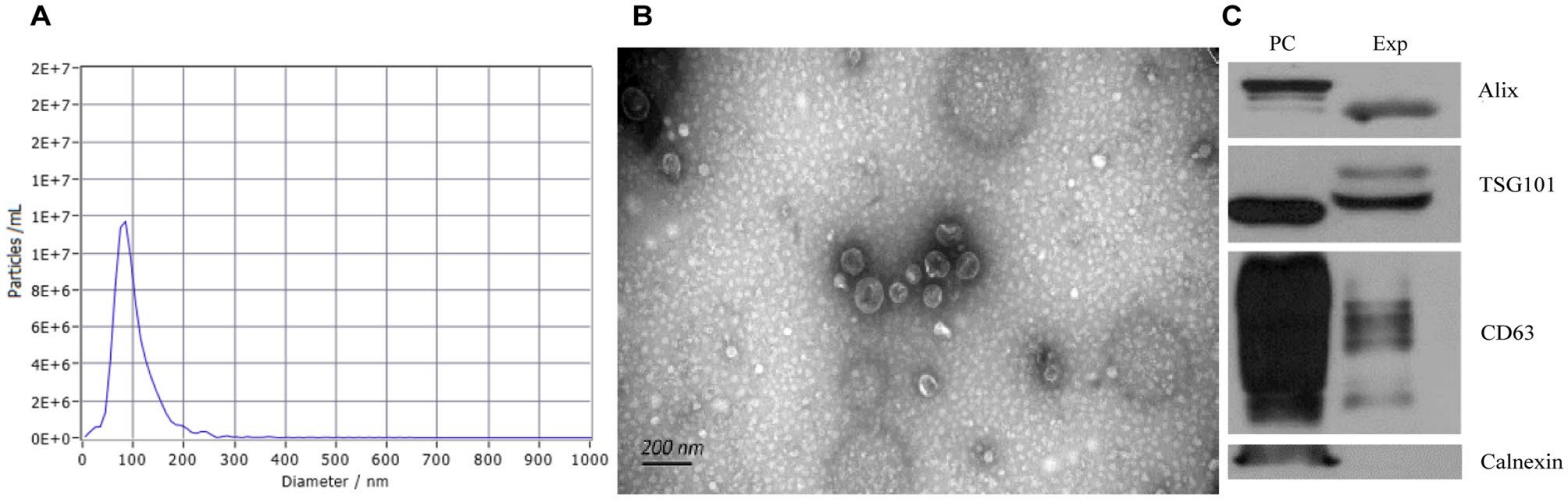
Characterization of exosomes derived from HCs and SUDs peripheral blood. (**A**) Representative nanoparticle tracking analysis report of exosomes from study participants. (**B**) Representative transmission electron micrograph images of exosomes derived from study participants, scale bar = 200 nm. (**C**) Western blot analysis showing the presence of three common positive exosomal markers (CD63, TSG101 and ALIX) and one negative exosomal marker (Calnexin) in exosomes isolated from study participants. *PC* positive control, *Exp* Experimental group.

We performed Nanoparticle Tracking Analysis (NTA), Transmission Electron Microscopy (TEM), and immunoblot analysis of protein markers specific for plasma-derived EVs to confirm that the nanoparticles we obtained were indeed exosomes. TEM showed that exosomes from HCs, HDPs, and MDPs all exhibited a cup-shaped morphology with diameters ranging from 50–150 nm (Fig. 1B), the median diameters were 99.4 ± 26.35, 100.8 ± 23.5, 99.5 ± 25.14, respectively. And the representative exosomal diameter was shown in Fig. 1A. There are no differences between health control and SUDs in both exosome size distribution (Figure S2A) and numbers (Figure S2B). Exosomes were also identified by western blotting for three commonly used exosomal markers (CD63, TSG101, and ALIX), and calnexin was used as a negative control (Fig. 1C). All these data indicated that the nanoparticles obtained were indeed exosomes.

### Overview of small RNA sequencing data and small RNA biotype mapping

A total of 630,546,931 raw reads were obtained after sequencing (Supplementary Table S1), which were generated from 30 small RNA libraries (10 replicates per group) derived from HCs, HDPs, and MDPs. After removing contaminant reads from the original data, a total of 240,754,247 clean reads were obtained from the 30 sequencing samples (Supplementary Table S2).

Reads obtained from sequencing were used for alignment and mapping to the human genome after adapter clipping and quality filtering. A total of 1299 distinct miRNAs were identified from all three groups from the combined raw read count data (Supplementary Table S2). The clean reads were then mapped to noncoding RNA databases, including the Rfam and miRBase databases, to annotate the classifications of the small RNAs. As shown in Supplementary Figure S3A, miRNAs were the most abundant exosomal RNA species in all the groups (64.27 ± 5.88%, 70.91 ± 5.28%, and 73.55 ± 4.82% in HCs, HDPs, and MDPs, respectively).

Length distribution analysis of the miRNAs showed that 20–24 nucleotides (nt) was the most frequent read length, with the peak at 22 nt, indicating that mature miRNAs were well enriched during the sequencing library preparation process (Supplementary Figure S3B). After identification of the conserved miRNAs, additional filtering was performed using miRDeep2 to identify potentially novel miRNAs. A total of 133 novel miRNAs were detected across the 30 libraries (Supplementary Table S2), and all displayed typical miRNA features at the genomic level. A representative readout of the predicted novel miRNAs is shown in Supplementary Figure S3C.

### DE-miRNAs and RT-qPCR validation in HCs and SUDs

**Figure 2:**
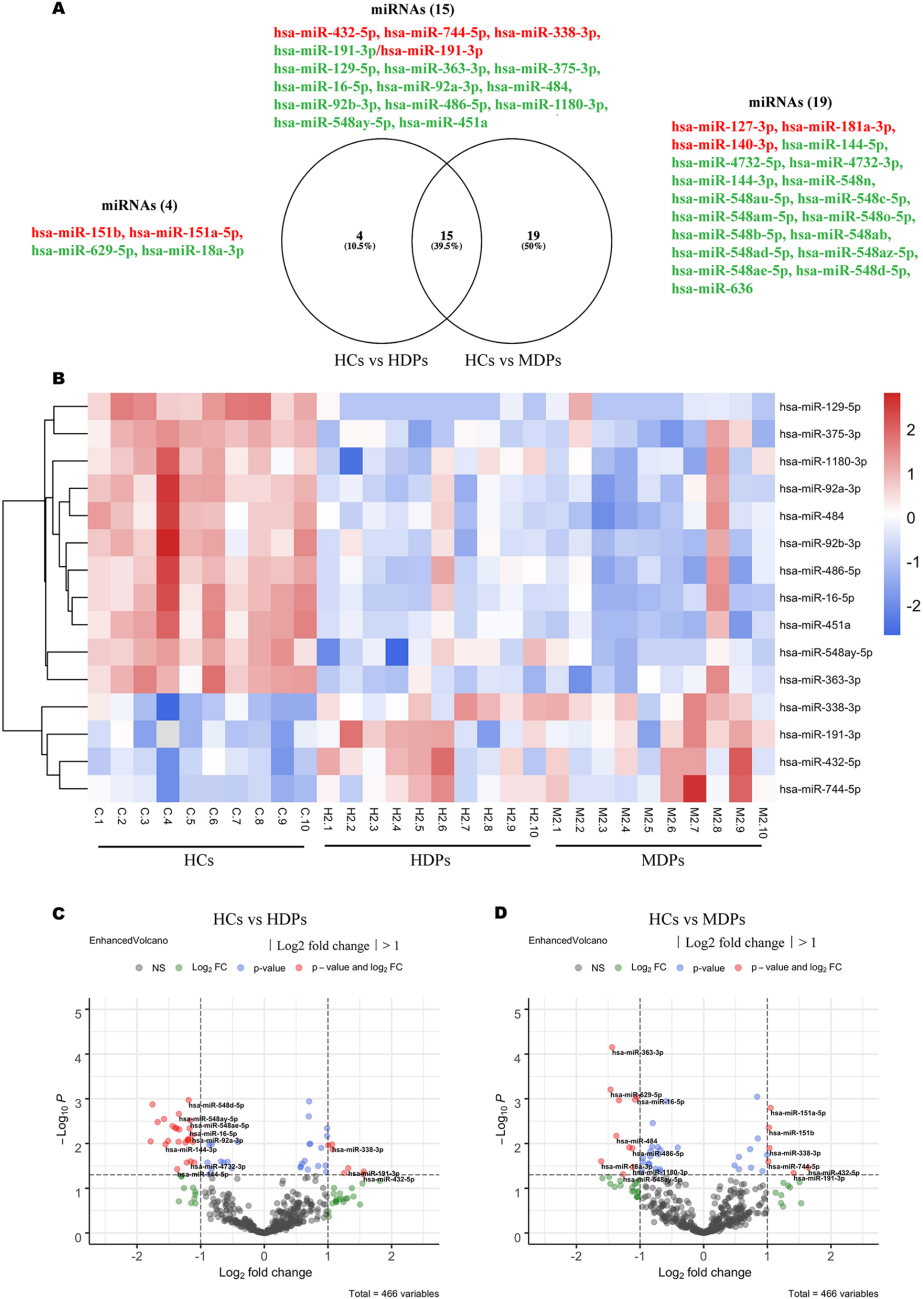
MiRNA expression profiles in exosomes isolated from SUDs and HCs. (**A**) Venn diagram showing overlapping miRNAs between DE-miRNAs from HCs vs HDPs and DE-miRNAs from HCs vs MDPs. (**B**) Hierarchical clustering analysis of miRNAs with altered expression among the three groups (*p* < 5e−02, fold change > 2). Red strip, high relative expression; blue strip, low relative expression; white strip, no change in gene expression. Color intensity reflects the degree of expression increase or decrease. (**C**) Volcano plot showing DE-miRNAs from HCs vs MDPs with various fold changes and *p*-values. Vertical line, fold change = 2 (log2 transformed); horizontal line, *p* = 5e−02 (−log10 transformed). Red dots, *p* < 5e−02, fold change > 2; green dots, change fold > 2, *p* < 5e−02; blue dots, *p* > 5e−02, fold change > 2; gray dots, insignificantly changed miRNAs. (**D**) Volcano plot showing DE-miRNAs from HCs vs HDPs with various fold changes and p-values. Vertical line, fold change = 2 (log2 transformed); horizontal line, *p* = 5e−02 (−log10 transformed). Red dots, *p* < 5e−02, fold change > 2; green dots, change fold > 2, *p* < 5e−02; blue dots, *p* > 5e−02, fold change > 2 ; gray dots, insignificantly changed miRNAs. *MDPs* Exosomes obtained from methamphetamine-dependent patients, *HDPs* Exosomes obtained from heroin-dependent patients, *HCs* Exosomes obtained from healthy controls, *SUDs* substance use disorders.

Differential expression analysis was performed using linear contrast in the DESeq2 RNA-seq, and controlled for 5% false-discovery rate (FDR) using the Benjamini–Hochberg method for each pairwise comparison. MiRNAs with an adjusted *p*-value < 5e−02, as determined by DESeq2, were assigned as being differentially expressed. We found 34 differentially expressed miRNAs between HCs and MDPs, 7 of which were significantly upregulated and 27 significantly downregulated (Fig. 2A, Supplementary Table S3a). Nineteen miRNAs were identified as DE-miRNAs between HCs and HDPs (Fig. 2A), 7 of which were markedly upregulated and 12 significantly downregulated (*p* < 5e−02) (Supplementary Table S3b). We also compared miRNAs expression between MDPs and HDPs, and identified 16 miRNAs with *p* value < 5e−02, but lost significance after FDR-adjusted. Fifteen miRNAs were identified as being differentially expressed in both the HCs vs MDPs and HCs vs MDPs comparisons.

Hierarchical clustering analysis was performed for these 15 DE-miRNAs (Fig. 2B) while volcano plots were used to assess the overall distribution of these DE-miRNAs in HCs vs HDPs (Fig. 2C) and HCs vs MDPs (Fig. 2D) comparisons.

**Figure 3:**
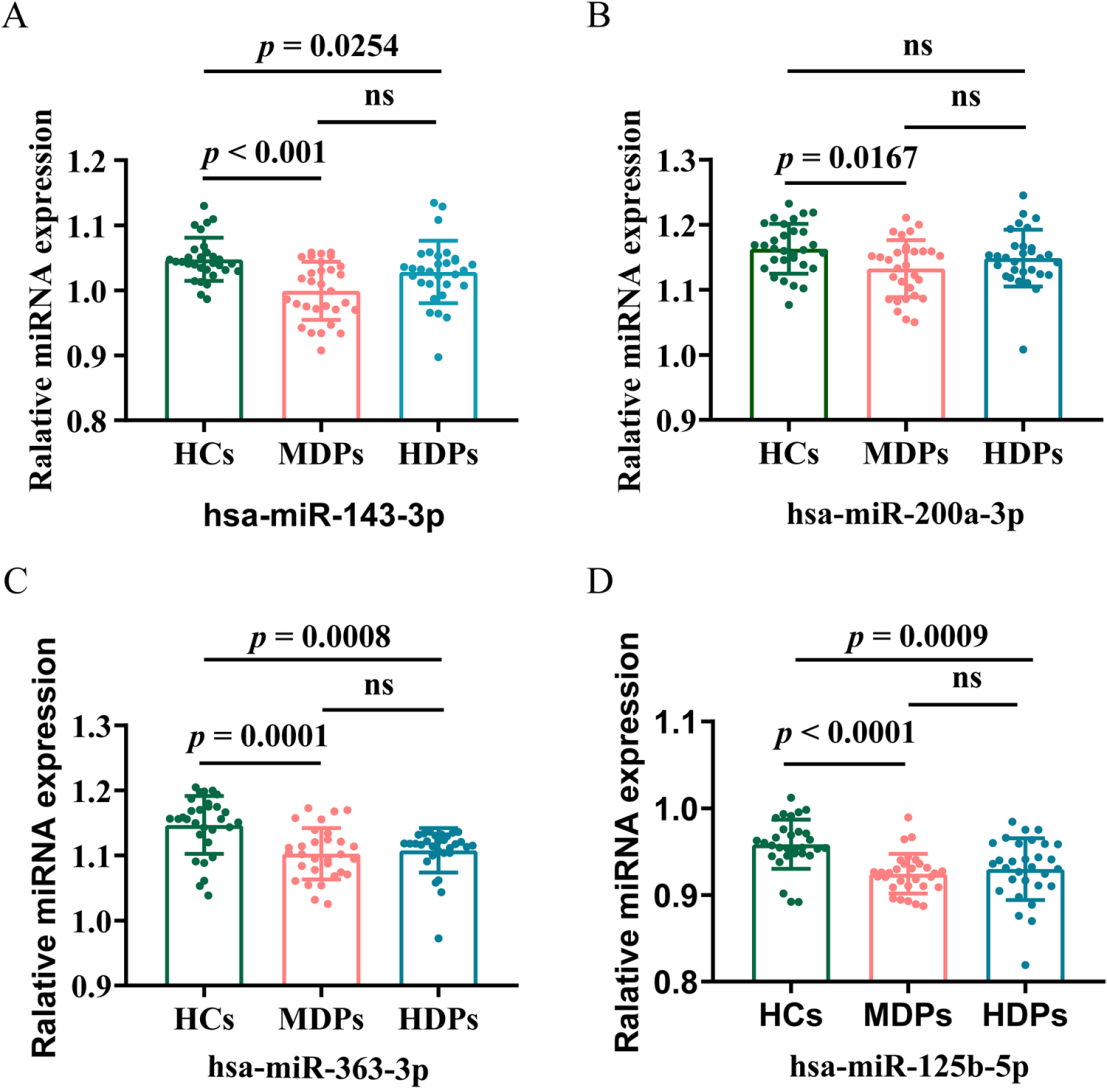
Expression level of 4 selected miRNAs assessed by real-time quantitative PCR. Relative expression level of miRNAs in the HCs, MDPs and HDPs groups, assessed by RT-qPCR; data are mean ± standard deviation (n = 30 in each group). (**A**) Relative expression level of hsa-miRNA-143-3p (**B**). Relative expression level of. (**C**). Relative expression level of hsa-miRNA-363-3p. (**D**). Relative expression level of hsa-miRNA-125-5p. *MDPs* Exosomes obtained from methamphetamine-dependent patients, *HDPs* Exosomes obtained from heroin-dependent patients, *HCs* Exosomes obtained from healthy controls.

Next, we recruited validation sets and used 30 samples for each group to isolate exosomes and validated the selected miRNAs identified by RNA-seq. We selected five of the DE-miRNAs for verification by qPCR assay, using 5′ nuclease probes, and normalized with U6 (Fig. 3), details of primer sequence were shown in Supplementary Table S4. Four of these showed a consistent trend with the RNA sequencing results for the MDP samples, and all were downregulated in MDPs when compared with HCs: hsa-miR-143-3p (Fig. 3A), hsa-miR-200a-3p (Fig. 3B), hsa-miR-363-3p (Fig. 3C), and hsa-miR-125b-5p (Fig. 3D) (all *p* < 5e−02); the change in hsa-miR-141-3p expression lost statistical significance in the qPCR validation (*p* > 5e−02). Three of the five hsa-miRNAs showed a consistent trend with the RNA sequencing results for the HDP samples: hsa-miR-143-3p, hsa-miR-363-3p, and hsa-miR-125b-5p (all *p* < 9.0e−04); the changes in hsa-miR-200a-3p and hsa-miR-141-3p expression level lost statistical significance in the qPCR validation (*p* > 5.0e−02).

### Correlation between exosomal DE-miRNAs and mood disturbance in SUDs

**Figure 4:**
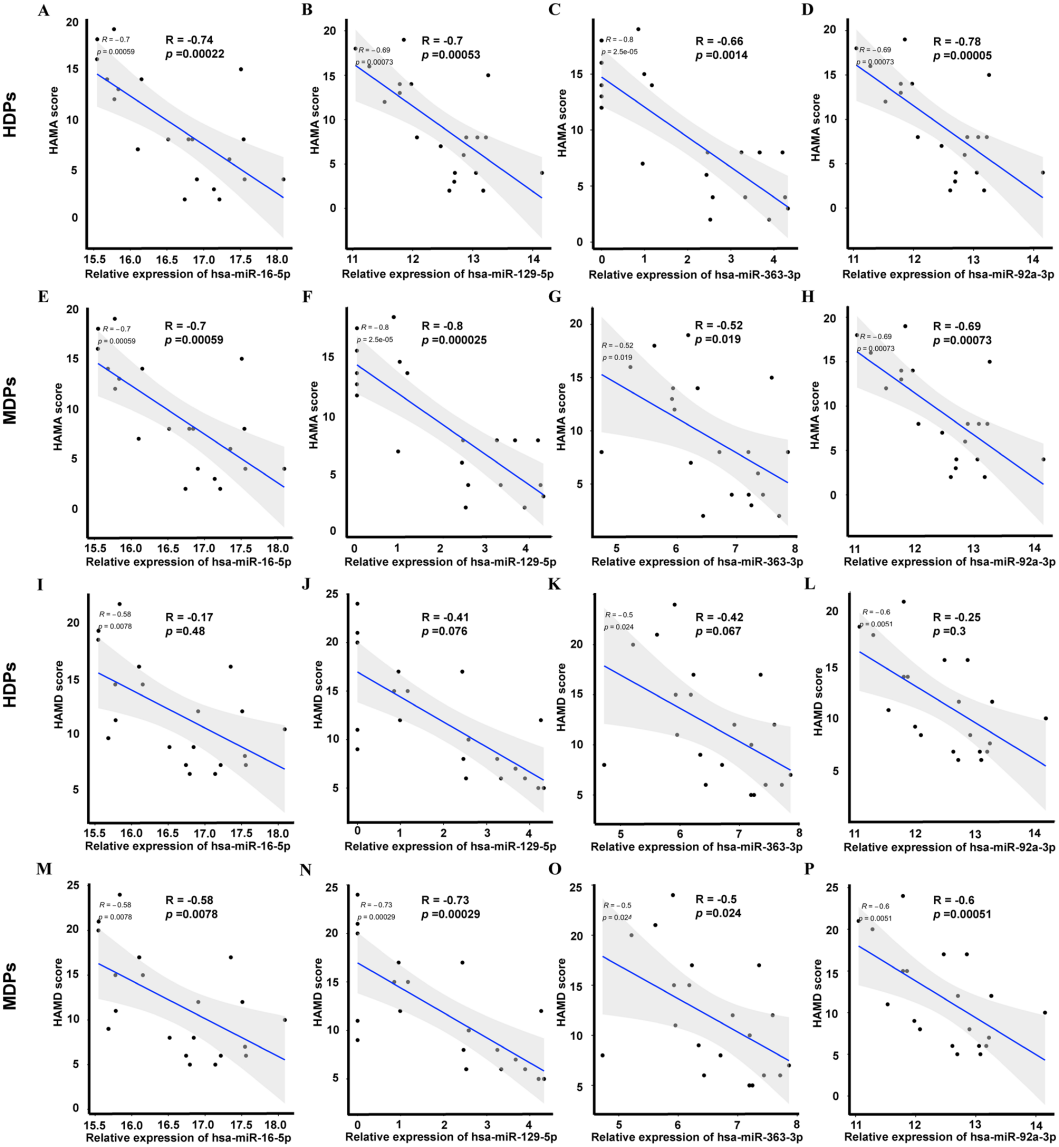
Correlation between exosomal DE-miRNAs and anxiety and depression in SUDs. (**A–H**) Correlation between exosomal DE-miRNAs and anxiety in SUDs. (**A**,**E**) correlation between hsa-miR-16-5p and HAM-A scores in HDPs and MDPs, respectively; (**B,F**) correlation between hsa-miR-129-5p and HAM-A scores in HDPs and MDPs, respectively; (**C**,**G**) correlation between hsa-miR-363-3p and HAM-A scores in HDPs and MDPs, respectively; (**D**,**H**) correlation between hsa-miR-92a-3p and HAM-A scores in HDPs and MDPs, respectively. (**I–P**) Correlation between exosomal DE-miRNAs and anxiety in SUDs. (**I**,**M**) correlation between hsa-miR-16-5p and HAM-D scores in HDPs and MDPs, respectively; (**J**,**N**) correlation between hsa-miR-129-5p and HAM-D scores in HDPs and MDPs, respectively; (**K**,**O**) correlation between miR-363-3p and HAMD in HDPs and MDPs, respectively; (**L**,**P**) correlation between miR-92a-3p and HAMD in HDPs and MDPs, respectively. The number on X-axis stands for reads calculated by TPM, the number on Y-axis stands for scores of HAM-A/HAM-D. *MDPs* Exosomes obtained from methamphetamine-dependent patients, *HDPs* Exosomes obtained from heroin-dependent patients, *HCs* Exosomes obtained from healthy controls, *SUDs* substance use disorders, *HAM-A* Hamilton Anxiety Rating Scale, *HAM-D* Hamilton Depression Rating Scale, *TPM* transcript per million.

We next used multiple linear regression analysis to assess whether a relationship existed between the expression of the DE-miRNAs and mood disturbances (the HAM-A/HAM-D values for the severity of anxiety and depression in SUDs). The results showed that all hsa-miR-16-5p (HDPs: *R* =  − 0.74, *p* = 2.2e−04, Fig. 4A; MDPs:* R* =  − 0.7, *p* = 5.9e−04, Fig. 4E), hsa-mi-129-5p (HDPs: *R* =  − 0.7, *p* = 5.3e−04, Fig. 4B; MDPs:* R* =  − 0.8, *p* = 2.5e−05, Fig. 4F), hsa-miR-363–39 (HDPs: *R* =  − 0.66, *p* = 1.4e−03, Fig. 4C; MDPs:* R* =  − 5.2, *p* = 1.9e−02, Fig. 4G), and hsa-miR-92a-3p (HDPs: *R* =  − 0.79, *p* = 5.0e−05, Fig. 4D; MDPs:* R* =  − 0.69, *p* = 7.3e−04, Fig. 4H) were negatively correlated with HAM-A scores in both HDPs and MDPs. All hsa-miR-16-5p (*R* =  − 0.58, *p* = 7.8e−03, Fig. 4I), hsa-miR-129-5p (*R* =  − 0.73, *p* = 2.9e−04, Fig. 4J), hsa-miR-363–39 (*R* =  − 0.5, *p* = 2.4e−02, Fig. 4K) and hsa-miR-92a-3p (*R* =  − 0.6, *p* = 5.1e−04, Fig. 4L) showed a negatively correlation with HAM-D scores in MDPs, while all these miRNAs exhibited no significance correlation with HAM-D scores in HDPs (all *p* > 5e−02, Fig. 4M–P).

### Potential regulatory roles of the identified DE-miRNAs

**Figure 5:**
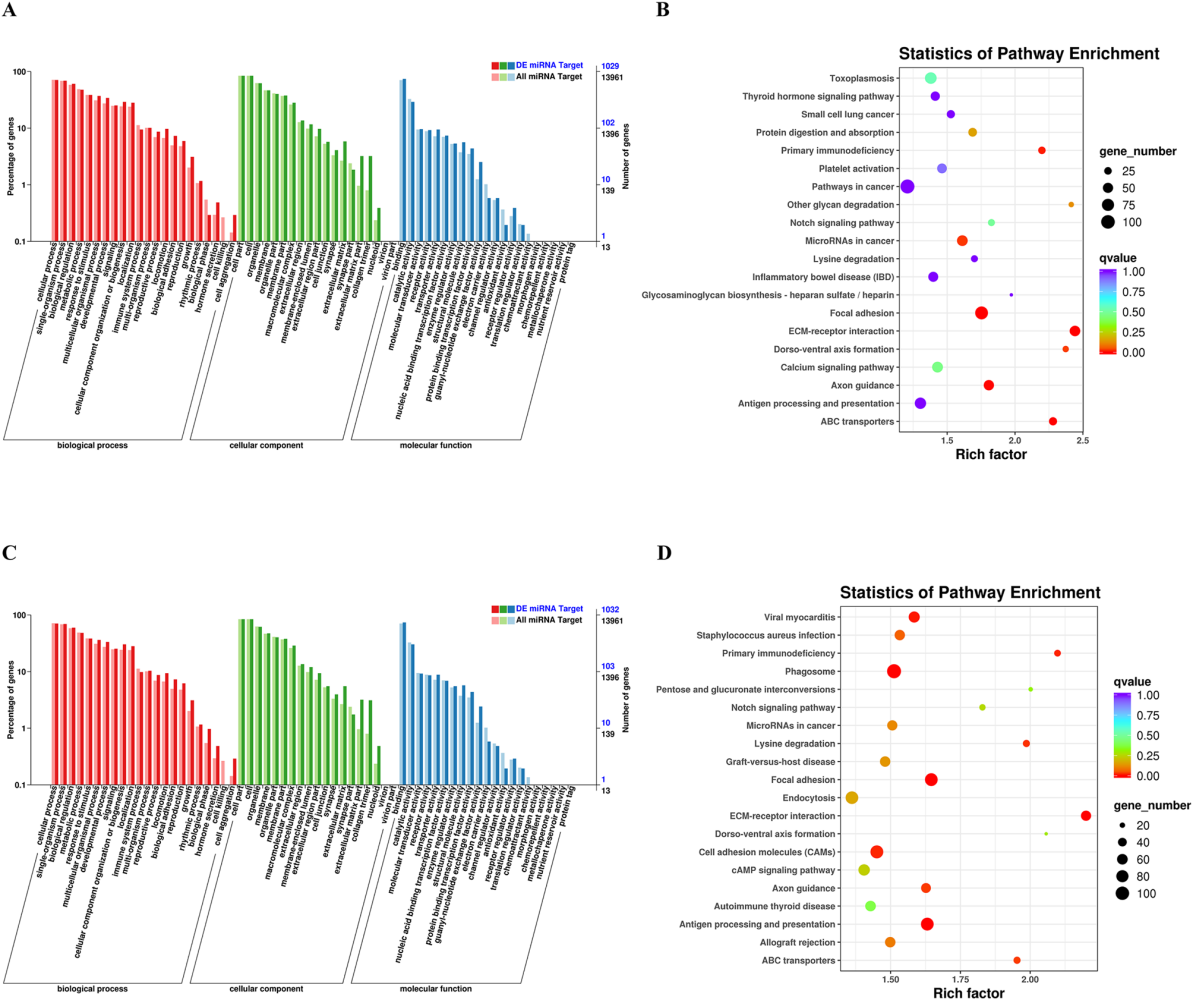
Major categories of GO terms and top 20 significantly enriched KEGG pathways regulated by candidate target genes of differentially expressed miRNAs. (**A**) Twenty most enriched GO terms in biological process, cellular component and molecular function for DE-miRNAs from HDPs vs HCs are listed. (**B**) KEGG pathways enriched for DE-miRNAs from HDPs vs HCs. Different color and diameter of the pathway dots represent significance level and gene number respectively. (**C**) Twenty most enriched GO terms in biological process, cellular component and molecular function for DE-miRNAs from HDPs vs HCs are listed. (**D**) KEGG pathways enriched for DE-miRNAs from MDPs vs HCs. Different color and diameter of the pathway dots represent significance level and gene number respectively. *GO* Gene Ontology, *KEGG* Kyoto Encyclopedia of Genes and Genomes, *DE* differentially expressed, *MDPs* Exosomes obtained from methamphetamine-dependent patients, *HDPs* Exosomes obtained from heroin-dependent patients, *HCs* Exosomes obtained from healthy controls.

We then performed GO and KEGG enrichment analyses to predict overlapping target genes and analyze the potential pathway involved in SUD-related anxiety and depression. For the comparison between HCs and HDPs (Fig. 5A), GO analysis indicated that the predicted targets were mainly enriched in nervous system (synapse and sympathetic nervous system development), cell mobility and proliferation (cell junction; cell adhesion; facal adhesion), and immune system (MHC class II protein complex; TAP complex) (Fig. 5B). The top 20 enriched GO terms of biological process, cellular component, and molecular function are displayed in Fig. 5C, which were predicted to play crucial roles in single-organism process, biological regulation, metabolic process. KEGG pathway analysis indicated that axon guidance, antigen processing and presentation, focal adhesion, and Notch signaling pathway were enriched in both MDPs and HDPs, implying that nervous system, cell mobility, and immune system werepresented among the enriched KEGG terms (Fig. 5D).

### Target interactome of the top 10 DE-miRNAs

**Figure 6:**
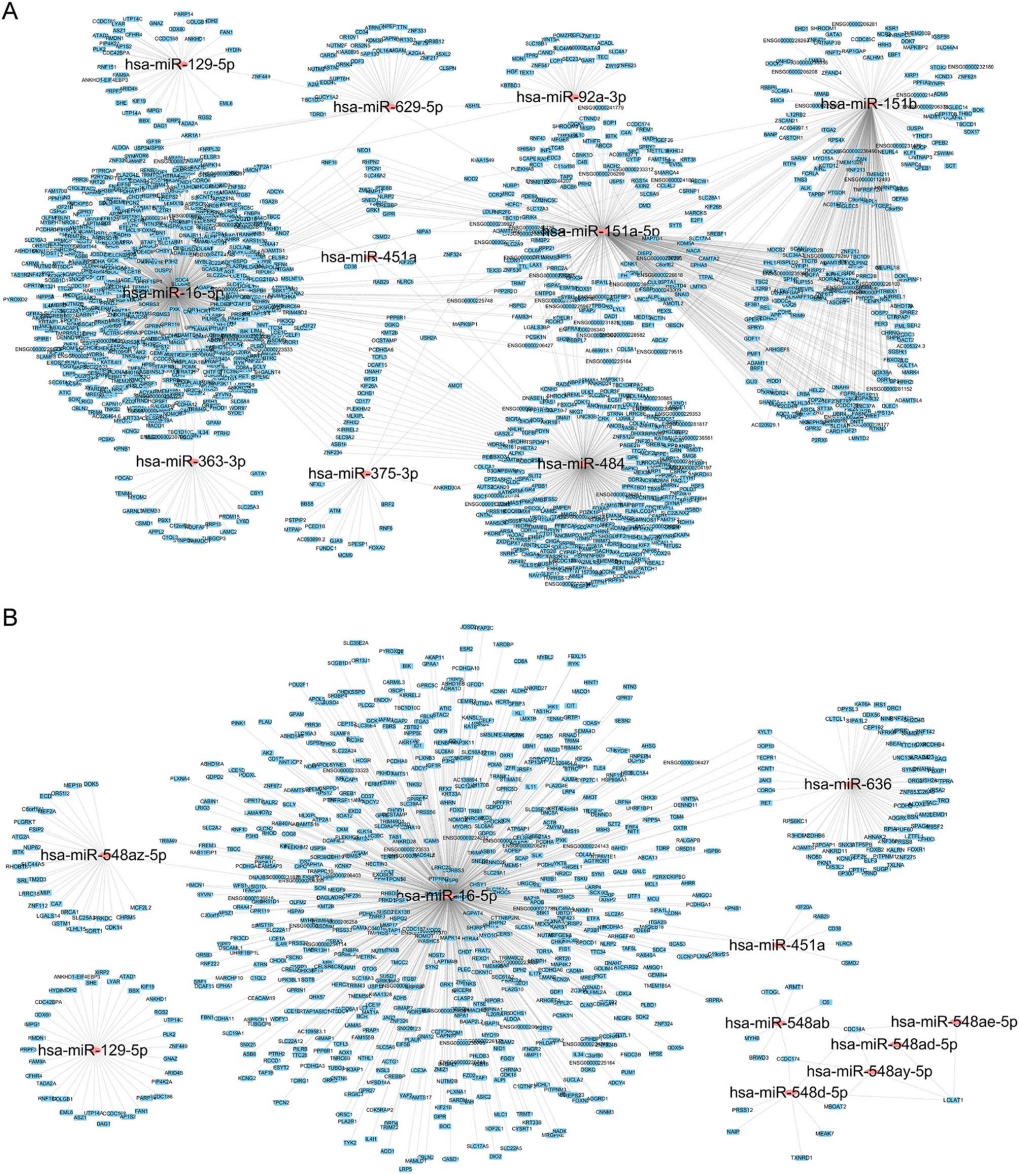
MiRNAs and mRNA interaction network analysis. (**A**) Top 10 DE-miRNAs from HCs vs HDPs along with the corresponding target genes were included in the network. Color corresponds to molecule type (miRNAs as red, mRNAs as blue). (**B**) Top 10 DE-miRNAs from HCs vs MDPs along with the corresponding target genes were included in the network. Color corresponds to molecule type (miRNAs as red, mRNAs as blue). *HDPs* Exosomes obtained from heroin-dependent patients, *MDPs* Exosomes obtained from methamphetamine-dependent patients, *HCs* Exosomes obtained from healthy controls.

MiRanda, TargetScan and miRDB were used to predict the target genes of the DE-miRNAs that overlapped between the HCs vs MDPs and HCs vs HDPs comparisons. MiRNA-gene interactions were assessed using Cytoscape. The top 10 DE-miRNAs (Supplementary Table S5a) between HCs and HDPs were connected by their respective mRNA targets, illustrating the network influenced by significantly differently regulated miRNAs (Fig. 6A). The respective mRNA targets of the top 10 DE-miRNAs (Supplementary Table S5b) between HCs and MDPs are depicted in Fig. 6B. The targeted 5299 genes were carried out overlapping with the aforementioned database on DE-miRNAs between HCs and patients with SUDs.

## Discussion

In the present study, we compared the expression profiles of plasma exosomal miRNAs derived from SUD patients and HCs. The expression level of 34 and 19 miRNAs were significantly altered in MDPs and HDPs, respectively, when compared with those of HCs. To further explore the functions of DE-miRNAs identified, GO and KEGG pathway analysis were performed for the predictive target genes. The results showed that target genes of the DE-miRNAs were mainly enriched in nervous system, cell mobility and proliferation, and immune system both in MDPs vs HCs and HDPs vs HCs. There are 16 DE-miRNAs were identified between MDPs and HDPs, but all these 16 miRNAs lost significant difference when adjusted by FDR.

Among the DE-miRNAs identified, part of them were reported to be involved in anxiety and depression. MiR-144-5p and miR-16-3p are involved in the response to mood stabilizer treatment^41^ and stress responses^42^. MiR-451a was altered in both serum and CSF from MDD patients^43^. And some other DE-miRNAs have been reported to be associated with neurological diseases, such as miR-181a and miR-484 were reported as mitomiRs involved in modulating mitochondrial function^44^, which played a vital role in neurodegenerative and psychological disease^45,46^.

We then compared the symptoms of anxiety and depression between SUD patients and HCs using HAM-A and HAM-D measures, and found that the HDPs had significantly higher scores in both the HAM-A and HAM-D measures, but MDPs lost statistical significance in the HAM-D. Notably, we also found statistically significant correlations between some miRNAs (hsa-miR-92a-3p, hsa-miR-363-3p, hsa-miR-16-5p, and hsa-miR-129-5p) and the total HAM-A scores, even after adjusting for years in education, in an ordinal logistic regression analysis. Hsa-miR-92a-3p, hsa-miR-363-3p, hsa-miR-16-5p, and hsa-miR-129-5p showed a significant correlation with total HAM-D scores in MDPs, but not in HDPs. It's worth noting that the expression of all the 4 DE-miRNAs aforementioned in MDPs displayed significantly negative correlations to HAM-D scores, while the HAM-D scores in the MDPs showed no difference when comparing to HCs. These findings implied that these four identified DE-miRNAs may have independent function in different type of SUDs, and have potential as biomarkers to assess the severity of anxiety and depression in SUD patients who are not diagnosed by scales.

Researches have shown that many of the targeted genes, such as those associated with neurotransmission/neuromodulation, including GABAergic, glutamatergic, dopaminergic, and serotonergic signaling, or with HPA axis functioning, are known to be aberrantly regulated in anxiety and depression^47^. Our study focused on exosomal miRNA profiles of SUD patients. We found that those 4 DE-miRNAs aforementioned were closely related to anxiety and depression. Gheysarzadeh et al. reported that the serum level of miR-16 was significantly downregulated in patients with anxiety and depression^42,48^, which was in agreement with our results. Shao et al. found the CSF miR-16, which has been suggested to be a biomarker for anxiety^49^, has a role in the pathogenesis of depression via affecting raphe SERT expression^50^, and play a role in the development of depressive-like behaviors in the hippocampus by targeting and downregulating *BNDF* expression^51^ via PI3K/Akt/mTOR pathway^52^. Furthermore, miR-16-5p was predicted to target to BDNF, NPY4R, GLUD1, and FKBP5, which are reported to be involved in the pathophysiology of anxiety and depression^53–57^. MiR-129-5p was predicted to negatively regulate neuropeptide FF receptor 2 (NPFFR2), which has been shown to activate the HPA axis and trigger anxiety- and depressive-like behaviors^58^. Nonetheless, to date, no systematic study has been carried out on circulating exosomal miRNA expression profiles of patients with SUDs and differential exosomal miRNAs in SUD patients may provided a plausible regulatory mechanism how peripheral molecular affecting central nerve system and behaviors.

In the present study, the circulating exosomal miRNA profiles of both MDPs and HDPs were investigated for the first time, leading to the identification of DE-miRNAs in MDPs and HDPs presenting with comorbid anxiety and depression. We explored the possible mechanism underlying the comorbidity of anxiety and depression associated with SUDs. Notably, GO and KEGG analyses consistently showed that these target genes were enriched for categories related to psychological disorder-associated processes, cell mobility, and the immune system. Molecular enrichment results have revealed that serine/threonine-protein kinases are involved in depressive-like behavior through the phosphorylation of eIF4E (eukaryotic initiation factor 4E) and consequent regulation of 5-HT neurotransmission^59^. Several cell adhesion molecules (CAMs), such as Np65 and NLGN-4, have been identified at neuromuscular junctions and synapses, where they recruit scaffolding proteins, neurotransmitter receptors, and synaptic vesicles^60,61^. They were indicated to be involved in regulating anxiety- and depressive-like behaviors, and proposed to have potential as transdiagnostic biomarkers. A different study showed that anxiety disorders are mediated through the modulation of miRNAs associated with the regulation of genes involved in axonal guidance^62^, a term that was also enriched in our biological analysis. DNA-Binding/Differentiation proteins, which was enriched in molecular analysis, have been shown to be connected with depression^63^.

Combined, our findings suggest that the identified DE-miRNAs may contribute to the biological functions of SUDs-derived exosomes via the regulation of the relevant pathways. Nevertheless, several limitations should be considered when interpreting our results. First, the power of this study was limited because of the relatively small sample size. To validate the sensitivity and specificity of the miRNAs as biomarkers for SUD-associated anxiety and depression, further research should be carried out with a larger sample size and in different centres. Second, the cross-sectional design of the study does not allow causal interpretations. Further investigation of DE-miRNAs and target pathways in longitudinal clinical studies or animal studies will help to validate the effects of these biomarkers.

## Conclusion

We identified exosomal miRNAs differentially expressed in patients with SUDs. The dysregulated miRNAs might be involved in the underlying pathophysiology of SUDs through biological pathways. Moreover, our results highlighted the importance of exosomal miRNAs as potential biomarkers for SUD patients suffering from depression and anxiety. Four DE-miRNAs were associated with anxiety in both MDPs and HDPs, but only associated with depression in MDPs, suggesting that these DE-miRNAs may be potential biomarkers for the diagnosis and treatment of anxiety and depression in SUDs.

## Materials and methods

### Ethics statement

All protocols and recruitment procedures described in this study were approved by the Research Ethics Committee of the First Affiliated Hospital of Kunming Medical University (2018-L-42) and conducted according to the tenets of the Declaration of Helsinki. All participants provided written informed consent before enrollment. Patients with SUDs were enrolled at the First Affiliated Hospital of Kunming Medicical University and the history of substance use was recorded by questionnaire. SUDs were diagnosed according to Fifth Edition of the Diagnostic and Statistical Manual of Mental Disorders (DSM-5) criteria. Healthy individuals were recruited from community sites. All the participants were male smokers with HIV(−), HBV(−) and HCV(−), then they were divided into a test set and a validation set. In the test set, 10 HCs and 20 gender- and age-matched SUD patients (10 HDPs and 10 MDPs) were recruited, poly drugs abusers were excluded. Thirty participants in each group were validated in the validation set.

### Sample collection and preparation

The demographics and substance use characteristics of the participants included in the study are presented in Table 1. Blood samples were collected from fasted SUD patients and HCs at 08:00 ~ 10:00 AM and using vacutainer blood collection tubes containing EDTA anticoagulant. The anticoagulant-treated blood samples were mixed by inverting the tube several times. Blood samples (10 ml were then centrifuged at 3000 rpm for 10 min at 20 °C. The upper layer containing plasma was transferred to a 2-ml eppendorf tube and centrifuged at 3000 × *g* for 15 min at 4 °C. Plasma from each sample was collected into a 4-ml eppendorf tube, immediately placed on dry ice, and then stored at − 80 °C until use.

### Scale administration

The Visual-Analogue Craving Scale (VAS) is used to measure the degree of drug craving using a 10 point scale, with 0 indicating “none at all” and 10 indicating “very much”. The scale records the subjective craving experienced by the study subjects at a specific time.

The HAM-A scale consists of 14 questions administered by the interviewer. Seven elements examine psychological stress and seven physical stress. The total score of the 14 items corresponds to the grade of stress severity: mild (score < 17), mild to moderate (score = 18–24), moderate to severe (score = 25–30), and severe (score > 30)^64^.

The HAM-D scale is a 24-item questionnaire administered by an interviewer that measures the severity of depressive symptoms. A total score above 20 is considered indicative of major depression^65^.

### Isolation of plasma exosomes

The exosomes was isolated by SEC (size exclution chromatography) methods as described previously with minor modifications^66^. Briefly, 1 mL of 0.8 μm-filtered blood plasma was 1.5-fold diluted with phosphate-buffered saline (PBS) and further purified using Exosupur columns (Echobiotech, China). The samples were then eluted with further 0.1 M PBS and a total of 2 mL eluate fractions were collected according to the manufacturer’s instructions. Fractions were concentrated to 200 μL by 100 kDa molecular weight cut-off Amicon Ultra spin filters (Merck, Germany). The resulted exosomes were resuspended in PBS and stored at − 80 °C or used for the downstream experiments.

### Western blot analysis

Exosomal protein concentrations were quantified using a BCA protein assay kit (Pierce, FL, USA) following the manufacturer’s protocol. Equal amounts of protein (20 μg) were vortexed in 5 × loading buffer and denatured at 95 °C for 5 min, separated by 10% SDS PAGE, and transferred onto polyvinylidene fluoride (PVDF) membranes by electroblotting. The membranes were then blocked with 5% BSA for 1 h at room temperature and incubated overnight at 4 °C with mouse anti-CD63 (1:200 dilution; Santa Cruz, CA, USA), mouse anti-ALIX (1:200 dilution; Santa Cruz, CA, USA), mouse anti-TSG101 (1:200 dilution; Santa Cruz, CA, USA), or rabbit anti-calnexin (1:1000 dilution; Promega, Madison, WI, USA) antibodies. After incubation with a specific secondary anti-mouse or -rabbit horseradish peroxidase-conjugated antibody (1:5000 dilution; GeneTex, USA) at room temperature for 1 h, the protein bands were detected using a chemiluminescence detection kit (Millipore Co., MA, USA) and scanned using the iBright FL1500 chemiluminescence imaging system (Thermo Fisher Scientific, USA).

### Transmission electron microscopy

For transmission electron microscopy (TEM) analysis, 10 μl of exosomal suspension was loaded on a copper mesh, incubated for 10 min at room temperature, and washed with distilled water. After adsorption, each sample was negatively stained with 10 μl of a 2% *(w/v*) uranyl acetate solution at room temperature for 1 min, and excess liquid was blotted with filter paper. Then, the samples were observed and photographed using a Hitachi H-7650 transmission electron microscope (Hitachi, Tokyo, Japan) operating at 80 kV.

### Nanoparticle tracking analysis

Vesicle suspensions with concentrations between 1 × 10^7^–10^9^/mL were examined using the ZetaView PMX 110 (Particle Metrix, Meerbusch, Germany) equipped with a 405 nm laser to determine the size and quantity of particles isolated^67^. A video of 60-s duration was taken with a frame rate of 30 frames/sec, and particle movement was analyzed using NTA software (ZetaView 8.02.28, https://www.particle-metrix.de).

### RNA preparation and library construction

Total RNA in the exosomes was extracted using the Qiagen miRNeasy Mini kit (Qiagen, CA, USA) following the manufacturer’s protocol. The concentration and quality of the RNA were determined by Agilent 2100 Bioanalyzer. Small RNA libraries were prepared using the QIAseq miRNA Library Kit (Qiagen, Frederick, MD) according to the manufacturer’s protocols. For each library, 1–500 ng of total RNA from each sample was used in all experimental procedures and index codes were added to attribute sequences to each sample. At last, library quality was assessed on the Agilent Bioanalyzer 2100 and qPCR. The clustering of the index-coded samples was performed on acBot Cluster Generation System using TruSeq PE Cluster Kitv3-cBot-HS (Illumina, San Diego, CA, USA) according to the manufacturer’s instructions. After cluster generation, the library preparations were sequenced on an Illumina Hiseq platform and paired-end reads were generated.

### Quantification and differential expression analysis of miRNA

Use Bowtie tools soft, The Clean Reads respectively with Silva database, GtRNAdb database, Rfam database and Repbase database sequence alignment, filter ribosomal RNA (rRNA), transfer RNA (tRNA), small nuclear RNA (snRNA), small nucleolar RNA (snoRNA) and other ncRNA and repeats. The remaining reads were used to detect known miRNA and new miRNA predicted by comparing with known miRNAs from miRbase and Human Genome (GRCh38), respectively. Read count for each miRNA was obtained from the mapping results, and Transcripts Per Million (TPM) was calculated.

### Prediction and functional analysis of miRNA-targeted genes

The mRNA targets of the DE-miRNAs were predicted using two databases (miRanda and RNAhybrid). DE-miRNAs were analyzed using a Venn diagram to identify overlapping genes. Only the miRNA-target gene interactions that showed overlap in two databases (miRanda and RNAhybrid) aformentioned were considered candidate targets. Gene–gene and a miRNA–target gene interaction networks were visualized using Cytoscape 3.0. The shared DE-miRNA-target interactions were also visualized using Cytoscape 3.0 (https://cytoscape.org). GO and KEGG (www.kegg.jp/kegg/kegg1.html) pathway enrichment analyses were performed to assess the potential functions of the identified target genes using topGO 3.12 (https://doi.org/10.18129/B9.bioc.topGO) and KOBAS (http://kobas.cbi.pku.edu.cn), respectively.

### Real-time quantitative PCR analysis of miRNAs

A few miRNAs identified through small RNA sequencing were validated by qPCR analysis of selected miRNA targets. The total RNA from exosomes was extracted using miRNeasy Mini kit (Qiagen, cat. No. 217004) according to the manufacturer’s protocol. The total RNA was then reverse transcribed to synthesize cDNA using PrimeScript RT reagent Kit (Perfect Real Time) (TAKARA, RR037A). The abundance of target gene expression was detected by TaqMan probe using real-time qPCR. 2 µL of cDNA was used as the template for each PCR reaction. The sequence of primers and probes were shown as Supplementary Table S1. Relative expression level was quantified by the ΔΔCt method.

### Statistical analysis

Statistical analysis was performed using SPSS version 24 (SPSS Inc., Chicago, IL, USA) and GraphPad Prism 8.0 (GraphPad Software, San Diego, CA, USA, https://www.graphpad.com). Quantitative data were expressed as means ± standard deviation (SD). Exosome concentrations were analyzed using the one-way ANOVA among three groups. An adjusted *p* value < 5e−02 was considered to indicate a statistically significant difference.

## Supplementary Information

Supplementary Information
