# Supplementary material for: Prognostic plasma exosomal microRNA biomarkers in patients with substance use disorders presenting comorbid with anxiety and depression
Source: Sci Rep. 2021 Mar 18;11:6271. doi: 10.1038/s41598-021-84501-5 (PMC7973758; doi:10.1038/s41598-021-84501-5)

*Tittle*: Prognostic plasma exosomal microRNA biomarkers in patients with substance use disorders presenting comorbid with anxiety and/or depression

Fengrong Chen^1,2^, Lei Zou^1, 4^, Yicong Dai^1^, Jiaxue Sun^1^, Cheng Chen^1^, Yongjin Zhang^1, 2^, Qingyan Peng^1,2^, Zunyue Zhang^1, 2^, Zhenrong Xie^1^, Hongjin Wu^1, 2^, Weiwei Tian^1, 2^, Xu Yu^1, 3^, Juehua Yu^1, 2^ & Kunhua Wang^1, 2, 3^

^1^NHC Key Laboratory of Drug Addiction Medicine, First Affiliated Hospital of Kunming Medical University, Kunming 650032, Yunnan, China

^2^Centre for Experimental Studies and Research, First Affiliated Hospital of Kunming Medical University, Kunming, Yunnan 650032, Yunnan, China

^3^Yunnan Institute of Digestive Disease, First Affiliated Hospital of Kunming Medical University, Kunming, Yunnan 650032, Yunnan, China

^4^Department of Organ Transplant, First Affiliated Hospital of Kunming Medical University, Kunming 650032, Yunnan, China

Fengrong Chen and Lei Zou contribute equally to this work.

Corresponence and requests for materials should be addressed to J.Y. (Email:; juehuayu@gmail.com) and K.W. (Email: [kunhuawang1@163.com](mailto:kunhuawang1@163.com)).

Supplementary Figure 1


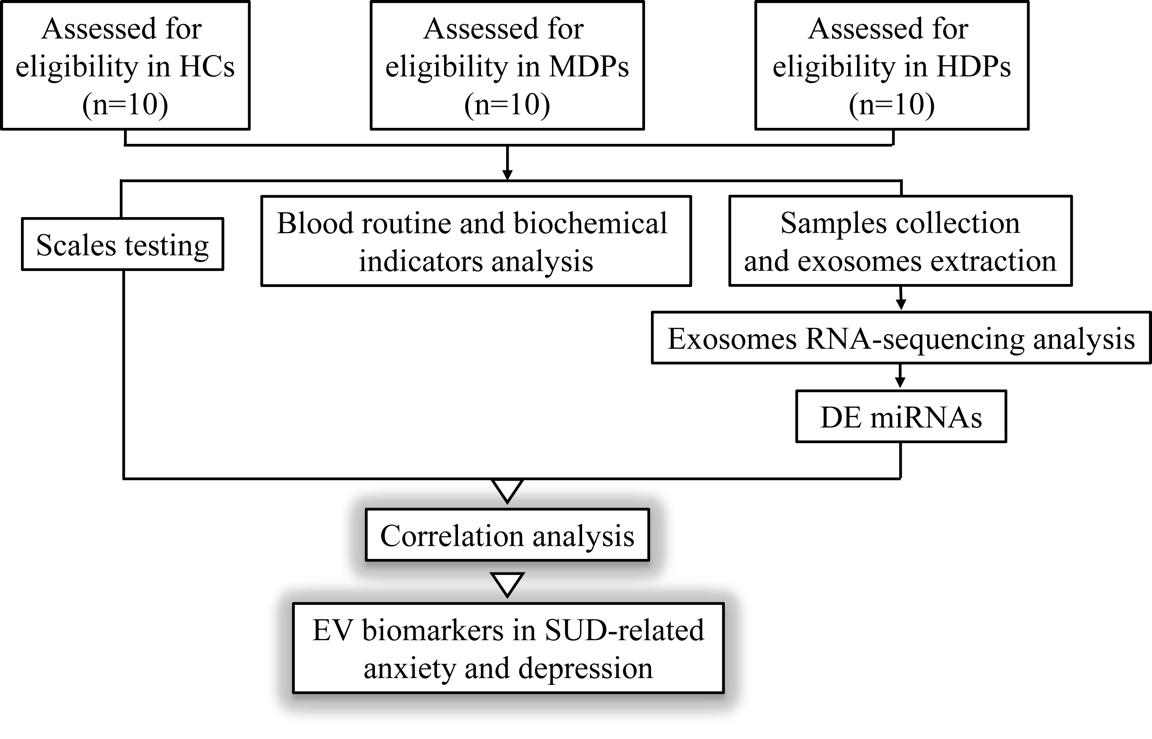


# Figure S1. Study design and workflow for experiment. Scale testing of anxiety and depression: The Hamilton Anxiety Rating Scale (HAMA) and Hamilton Depression Rating Scale (HAMD) were used to assess anxiety and depression. The scores corresponded to the grade of anxiety and depression severity. Samples collection and exosomes extraction: Peripheral blood from healthy controls (HCs), heroin use dependents (HDPs) and methamphetamine use dependents (MDPs) (n = 10 each group) were collected and exosomal miRNAs were extracted. RNA-Sequencing and Analysis: Total RNA in the exosomes was extracted, small RNA libraries were prepared, and then were sequenced and paired-end reads were generated. Quantification and differential expression analysis of miRNAs (DE-miRNAs) were conducted last. Correlation between exosomal DE-miRNAs and mood disturbance: Linear regression analysis to assess whether a relationship existed between the expression of the DE-miRNAs and mood disturbances (anxiety and depression).

Supplementary Figure 2


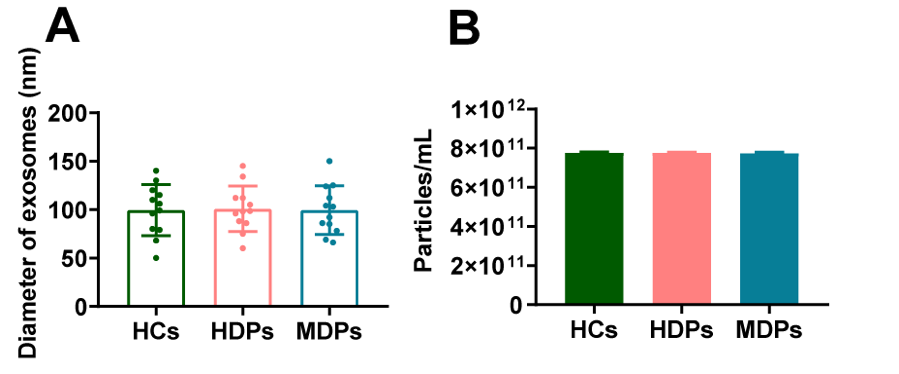


**Figure S2. Size distribution and numbers of exosomes derived from peripheral blood. A.** Statistics of diameters of exosomes from HCs, MDPs and HDPs samples. **B**. Numbers of exosomes from all HCs, MDPs and HDPs samples.

MDPs: Exosomes obtained from methamphetamine-dependent patients; HDPs: Exosomes obtained from heroin-dependent patients; HCs: Exosomes obtained from healthy controls.

Supplementary Figure 3


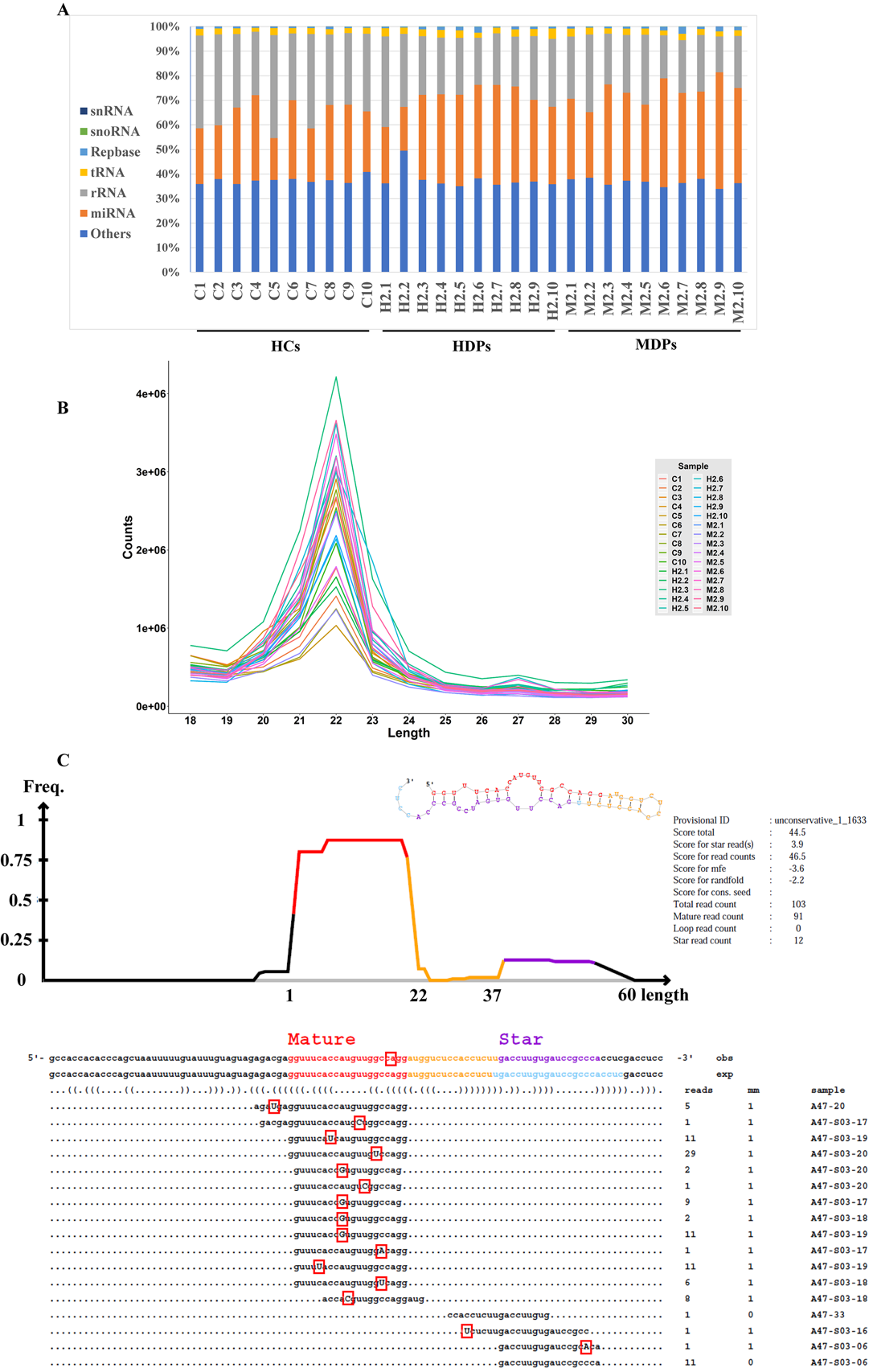


**Figure S3. Small RNA classification in exosomes derived from peripheral blood**

**A**. Statistics of each annotation type mapped to human genome and noncoding RNA databases. **B**. Length distribution of all known miRNAs in all HCs, MDPs and HDPs samples. **C**. A representative novel miRNA predicted by miRDeep2. Upper right table shows the miRDeep2 scores and the read count for each component of the putative miRNA. Mismatched nucleotides are indicated by uppercase letters (showed in red rectangle). mm, number of mismatches. MDPs: Exosomes obtained from methamphetamine-dependent patients; HDPs: Exosomes obtained from heroin-dependent patients; HCs: Exosomes obtained from healthy controls.

| **Table S1. Hematological parameters and biochemical indicators of study participants** | | | | | |
| --- | --- | --- | --- | --- | --- |
| Parameters | HCs  (M ± SD，n = 10) | HDPs (M ± SD，n = 10) | Adjusted *p* value （HC vs HDPs） | MDPs (M ± SD，n = 10) | Adjusted *p* value （HC vs MDPs） |
| WBC (10^9/L) | 5.2±0.49 | 5.68±0.35 | **0.0462** | 5.4±0.42 | 0.5867 |
| HCT (L/L) | 0.45±0.03 | 0.49±0.04 | **0.04** | 0.51±0.03 | **0.004** |
| MCHC (g/L) | 342.6±5.84 | 325.7±8.26 | **0.0002** | 323.63±9.58 | **< 0.0001** |
| NEUT# (10^9/L) | 3.34±1.34 | 5.32±1.3 | **0.0089** | 3.87±1.47 | 0.6926 |
| BASO# (10^9/L) | 0.03±0.02 | 0.06±0.03 | 0.0513 | 0.06±0.02 | **0.0153** |
| BASO% | 0.41±0.31 | 0.76±0.33 | **0.012** | 0.86±0.3 | **0.012** |
| TP | 76.05±4.58 | 89.74±7.1 | **< 0.0001** | 90.68±4.02 | **< 0.0001** |
| ALB | 48.83±2.11 | 54.73±2.82 | **< 0.0001** | 55.45±2.09 | **< 0.0001** |
| GLB | 27.22±3.95 | 34.71±5.13 | **0.002** | 35.23±3.71 | **0.0019** |
| AST/ALT | 1.29±0.59 | 0.84±0.12 | **0.0301** | 0.73±0.18 | **0.0106** |
| PAB | 284.22±49.57 | 321.1±39.64 | 0.154 | 349.63±37.56 | **0.0098** |
| Na (mmol/L) | 141.5±2.12 | 145.23±1.73 | **0.0014** | 146.25±2.44 | **0.0002** |
| Mg (mmol/L) | 0.81±0.09 | 0.92±0.04 | **0.0225** | 0.93±0.11 | **0.0164** |
| P (mmol/L) | 1.16±0.23 | 1.39±0.11 | **0.0183** | 1.26±0.15 | 0.447 |
| C3 (g/L) | 0.97±0.12 | 1.24±0.12 | **0.0001** | 1.27±0.13 | **< 0.0001** |
| **Abbreviations:** M, mean; SD, standard deviation; MDPs, methamphetamine-dependent patients; HDPs, heroin-dependent patients; HCs, healthy controls. | | | | | |

| **Table S2. Summary of small RNA sequencing statistics** | | | | | | | | |
| --- | --- | --- | --- | --- | --- | --- | --- | --- |
| **Sample** | **Number of reads** | | | | |  | **Number of miRNA** | |
|  | **Raw_reads** | **Length trimmed** | **Quality filtered** | **N trimmed** | **Clean_reads** |  | **Known** | **Putative novel** |
| **All samples** | 630546931 | 240759546 | 240759546 | 240754247 | 240754247 |  | 1299 | 133 |
| HC1 | 28951357 | 6993344 | 6993344 | 6993340 | 6993340 |  | 612 | 22 |
| HC2 | 24323530 | 5710597 | 5710597 | 5710596 | 5710596 |  | 552 | 21 |
| HC3 | 25745696 | 8515690 | 8515690 | 8515689 | 8515689 |  | 598 | 24 |
| HC4 | 23373769 | 8358123 | 8358123 | 8358122 | 8358122 |  | 662 | 26 |
| HC5 | 20854033 | 5018514 | 5018514 | 5018512 | 5018512 |  | 436 | 24 |
| HC6 | 23011904 | 8686982 | 8686982 | 8686979 | 8686979 |  | 646 | 27 |
| HC7 | 19440842 | 4939177 | 4939177 | 4939170 | 4939170 |  | 468 | 16 |
| HC8 | 23087794 | 8468396 | 8468396 | 8468392 | 8468392 |  | 592 | 31 |
| HC9 | 25512976 | 7368351 | 7368351 | 7368347 | 7368347 |  | 618 | 21 |
| HC10 | 21970702 | 7339262 | 7339262 | 7339252 | 7339252 |  | 614 | 24 |
| HDP1 | 18770486 | 6947738 | 6947738 | 6947738 | 6947738 |  | 438 | 12 |
| HDP2 | 19452501 | 8092496 | 8092496 | 8090758 | 8090758 |  | 360 | 10 |
| HDP3 | 30102744 | 13926937 | 13926937 | 13926937 | 13926937 |  | 510 | 21 |
| HDP4 | 20519446 | 9806286 | 9806286 | 9806286 | 9806286 |  | 528 | 19 |
| HDP5 | 20054726 | 8762403 | 8762403 | 8762403 | 8762403 |  | 655 | 21 |
| HDP6 | 20632304 | 9825993 | 9825993 | 9825990 | 9825990 |  | 597 | 23 |
| HDP7 | 20027723 | 10135235 | 10135235 | 10135235 | 10135235 |  | 479 | 15 |
| HDP8 | 17216335 | 6483377 | 6483377 | 6483376 | 6483376 |  | 489 | 23 |
| HDP9 | 20277871 | 7682860 | 7682860 | 7682860 | 7682860 |  | 501 | 18 |
| HDP10 | 18488431 | 7421336 | 7421336 | 7421334 | 7421334 |  | 486 | 14 |
| MDP1 | 18686291 | 7814755 | 7814755 | 7814753 | 7814753 |  | 516 | 20 |
| MDP2 | 19003637 | 4690820 | 4690820 | 4690194 | 4690194 |  | 380 | 10 |
| MDP3 | 19159441 | 8429956 | 8429956 | 8429955 | 8429955 |  | 494 | 18 |
| MDP4 | 19387191 | 7719314 | 7719314 | 7719314 | 7719314 |  | 432 | 9 |
| MDP5 | 17760736 | 6064302 | 6064302 | 6064302 | 6064302 |  | 442 | 13 |
| MDP6 | 18348826 | 8840170 | 8840170 | 8839600 | 8839600 |  | 560 | 21 |
| MDP7 | 19776356 | 9027219 | 9027219 | 9026441 | 9026441 |  | 591 | 36 |
| MDP8 | 19283791 | 8803941 | 8803941 | 8803140 | 8803140 |  | 590 | 27 |
| MDP9 | 19239913 | 10264909 | 10264909 | 10264909 | 10264909 |  | 664 | 33 |
| MDP10 | 18085579 | 8621063 | 8621063 | 8620323 | 8620323 |  | 438 | 26 |
| **Abbreviations:** MDPs, methamphetamine-dependent patients; HDPs, heroin-dependent patients; HCs, healthy controls. | | | | | | | | |

| **Table S3a. Details of DE-miRNAs from HCs vs HDPs** | | | | | | | |
| --- | --- | --- | --- | --- | --- | --- | --- |
| **Gene_id** | **CT (log2)** | **CT (SD)** | **HDP (log2)** | **HDP (SD)** | **Fold Change (log2)** | ***p* value** | **FDR** |
| hsa-miR-129-5p | 3.368 | 0.803 | 0.291 | 0.624 | -3.077 | 3.01E-08 | 1.40E-05 |
| hsa-miR-363-3p | 7.208 | 0.434 | 5.769 | 0.369 | -1.438 | 3.02E-07 | 7.04E-05 |
| hsa-miR-629-5p | 6.732 | 0.502 | 5.269 | 0.503 | -1.463 | 4.01E-06 | 0.000623126 |
| hsa-miR-375-3p | 7.056 | 0.698 | 3.781 | 1.337 | -3.274 | 9.11E-06 | 0.000849115 |
| hsa-miR-16-5p | 17.219 | 0.428 | 16.172 | 0.33 | -1.047 | 1.15E-05 | 0.000890649 |
| hsa-miR-92a-3p | 13.043 | 0.443 | 11.965 | 0.385 | -1.078 | 1.80E-05 | 0.001049064 |
| hsa-miR-451a | 11.901 | 0.573 | 10.57 | 0.297 | -1.33 | 1.62E-05 | 0.001080192 |
| hsa-miR-151a-5p | 6.649 | 0.484 | 7.7 | 0.322 | 1.052 | 3.44E-05 | 0.001600742 |
| hsa-miR-151b | 5.928 | 0.523 | 6.954 | 0.377 | 1.026 | 0.00011 | 0.004429157 |
| hsa-miR-484 | 7.453 | 0.722 | 6.083 | 0.557 | -1.37 | 0.00019 | 0.006735895 |
| hsa-miR-92b-3p | 6.689 | 0.678 | 5.52 | 0.497 | -1.169 | 0.00042 | 0.012187598 |
| hsa-miR-338-3p | 5.288 | 0.621 | 6.321 | 0.449 | 1.033 | 0.00057 | 0.012582637 |
| hsa-miR-486-5p | 14.308 | 0.55 | 13.189 | 0.637 | -1.119 | 0.00055 | 0.012814644 |
| hsa-miR-18a-3p | 2.475 | 0.976 | 0.866 | 0.958 | -1.61 | 0.00156 | 0.025036787 |
| hsa-miR-744-5p | 6.887 | 0.571 | 7.903 | 0.65 | 1.017 | 0.00162 | 0.025149073 |
| hsa-miR-1180-3p | 5.002 | 0.521 | 3.886 | 0.827 | -1.116 | 0.00253 | 0.033695503 |
| hsa-miR-432-5p | 5.629 | 0.855 | 7.273 | 1.194 | 1.644 | 0.00266 | 0.034478576 |
| hsa-miR-191-3p | 1.968 | 0.915 | 3.381 | 1.015 | 1.413 | 0.00431 | 0.045645691 |
| hsa-miR-548ay-5p | 4.452 | 0.347 | 3.185 | 1.076 | -1.267 | 0.0047 | 0.0486254 |
| **Abbreviations:** MDPs, methamphetamine-dependent patients; HDPs, heroin-dependent patients; HCs, healthy controls; C stands for Cycle and T stands for threshold; FDR: false discovery rate. | | | | | | | |

| **Table S3b. Details of DE miRNAs from HCs vs MDPs** | | | | | | | | |
| --- | --- | --- | --- | --- | --- | --- | --- | --- |
| **Gene_id** | **CT (log2)** | **CT (SD)** | **MDP (log2)** | **MDP (SD)** | **Fold Change (log2)** | ***p* value** | **FDR** | |
| hsa-miR-548d-5p | 4.518 | 0.344 | 3.329 | 0.494 | -1.189 | 1.15E-05 | 0.001071406 | |
| hsa-miR-451a | 11.901 | 0.573 | 10.143 | 0.688 | -1.757 | 8.62E-06 | 0.00133823 | |
| hsa-miR-129-5p | 3.368 | 0.803 | 0.721 | 1.018 | -2.647 | 5.77E-06 | 0.001344041 | |
| hsa-miR-548ay-5p | 4.452 | 0.347 | 3.109 | 0.519 | -1.343 | 4.67E-06 | 0.002177273 | |
| hsa-miR-636 | 2.072 | 0.665 | 0.496 | 0.648 | -1.576 | 4.25E-05 | 0.002830588 | |
| hsa-miR-548ae-5p | 4.51 | 0.32 | 3.345 | 0.57 | -1.165 | 5.86E-05 | 0.003033191 | |
| hsa-miR-548az-5p | 4.154 | 0.573 | 2.478 | 0.8 | -1.676 | 5.69E-05 | 0.003316864 | |
| hsa-miR-548ad-5p | 4.361 | 0.422 | 2.919 | 0.734 | -1.442 | 8.75E-05 | 0.004079763 | |
| hsa-miR-92b-3p | 6.689 | 0.678 | 5.291 | 0.599 | -1.398 | 0.000123416 | 0.004424006 | |
| hsa-miR-16-5p | 17.219 | 0.428 | 16.045 | 0.596 | -1.174 | 0.00010835 | 0.004590088 | |
| hsa-miR-548ab | 4.018 | 0.527 | 2.638 | 0.699 | -1.38 | 0.000118736 | 0.004610904 | |
| hsa-miR-548b-5p | 4.15 | 0.466 | 2.814 | 0.708 | -1.336 | 0.000145823 | 0.00485382 | |
| hsa-miR-92a-3p | 13.043 | 0.443 | 11.906 | 0.621 | -1.137 | 0.00022427 | 0.006531866 | |
| hsa-miR-548o-5p | 4.165 | 0.52 | 2.971 | 0.662 | -1.194 | 0.000325438 | 0.007981805 | |
| hsa-miR-548am-5p | 4.155 | 0.519 | 2.95 | 0.681 | -1.205 | 0.000357577 | 0.008331552 | |
| hsa-miR-548c-5p | 4.165 | 0.52 | 2.971 | 0.662 | -1.194 | 0.000325438 | 0.008425238 | |
| hsa-miR-486-5p | 14.308 | 0.55 | 12.796 | 0.904 | -1.512 | 0.000414913 | 0.008788614 | |
| hsa-miR-484 | 7.453 | 0.722 | 5.666 | 1.069 | -1.787 | 0.000480524 | 0.008956972 | |
| hsa-miR-548au-5p | 4 | 0.528 | 2.653 | 0.795 | -1.347 | 0.000411564 | 0.009132801 | |
| hsa-miR-548n | 3.94 | 0.6 | 2.818 | 0.577 | -1.122 | 0.000470887 | 0.009143048 | |
| hsa-miR-363-3p | 7.208 | 0.434 | 5.969 | 0.755 | -1.239 | 0.000467219 | 0.009466257 | |
| hsa-miR-144-3p | 8.037 | 0.939 | 6.485 | 0.715 | -1.553 | 0.000671335 | 0.010428069 | |
| hsa-miR-338-3p | 5.288 | 0.621 | 6.353 | 0.515 | 1.065 | 0.000608906 | 0.010509266 | |
| hsa-miR-375-3p | 7.056 | 0.698 | 3.759 | 2.152 | -3.297 | 0.000776987 | 0.010972001 | |
| hsa-miR-140-3p | 8.013 | 0.329 | 9.018 | 0.658 | 1.005 | 0.000800615 | 0.010973131 | |
| hsa-miR-181a-3p | 4.114 | 0.61 | 5.186 | 0.619 | 1.072 | 0.001045647 | 0.013535314 | |
| hsa-miR-4732-3p | 5.381 | 0.44 | 4.222 | 0.862 | -1.159 | 0.002145352 | 0.024993356 | |
| hsa-miR-4732-5p | 6.381 | 0.799 | 5.163 | 0.737 | -1.218 | 0.002347148 | 0.026677345 | |
| hsa-miR-1180-3p | 5.002 | 0.521 | 3.902 | 0.807 | -1.101 | 0.002406823 | 0.026704276 | |
| hsa-miR-191-3p | 1.968 | 0.915 | 3.287 | 0.861 | 1.319 | 0.003832873 | 0.035722373 | |
| hsa-miR-144-5p | 9.064 | 1.116 | 7.696 | 0.598 | -1.368 | 0.004261646 | 0.037470321 | |
| hsa-miR-127-3p | 3.51 | 0.833 | 5.072 | 1.267 | 1.562 | 0.005090147 | 0.042357298 | |
| hsa-miR-744-5p | 6.887 | 0.571 | 8.142 | 1.067 | 1.255 | 0.005600471 | 0.045786306 | |
| hsa-miR-432-5p | 5.629 | 0.855 | 7.183 | 1.29 | 1.554 | 0.006027813 | 0.048430362 | |
| **Abbreviations:** MDPs, methamphetamine-dependent patients; HDPs, heroin-dependent patients; HCs, healthy controls; C stands for Cycle and T stands for threshold; FDR: false discovery rate. | | | | | | | |  |

| **Table S3c. Details of DE miRNAs from HDPs vs MDPs** | | | |
| --- | --- | --- | --- |
| **Gene_id** | **Fold Change**  **(-log2)** | ***P* value** | **FDR** |
| hsa-miR-16-2-3p | -1.207073182 | 0.011724526 | 0.910604826 |
| hsa-miR-6803-3p | -1.076183302 | 0.034545469 | 1.006136792 |
| hsa-miR-144-3p | -0.911611232 | 0.004050644 | 0.943800159 |
| hsa-miR-365a-5p | -0.88938029 | 0.005387853 | 0.836913171 |
| hsa-miR-6859-5p | -0.657439004 | 0.045884092 | 1.018189861 |
| hsa-miR-4429 | 0.606188671 | 0.005963566 | 0.694755417 |
| hsa-miR-143-3p | 0.60925711 | 0.028955726 | 1.124447358 |
| hsa-miR-95-3p | 0.62950741 | 0.000869695 | 0.405277831 |
| hsa-miR-629-5p | 0.64606965 | 0.030677931 | 1.021136833 |
| hsa-miR-3173-5p | 0.706337509 | 0.029514929 | 1.057996688 |
| hsa-miR-324-3p | 0.76989352 | 0.006123467 | 0.570707136 |
| hsa-miR-511-5p | 0.825169972 | 0.042996135 | 1.054536774 |
| hsa-miR-3064-5p | 0.995528906 | 0.036690387 | 1.005748267 |
| hsa-miR-542-3p | 1.182057527 | 0.041469091 | 1.073588701 |
| hsa-miR-200a-3p | 1.188228527 | 0.031076137 | 0.965431997 |
| hsa-miR-141-3p | 1.223116384 | 0.02865614 | 1.213978287 |
| **Abbreviations:** MDPs, methamphetamine-dependent patients; HDPs, heroin-dependent patients; HCs, healthy controls; C stands for Cycle and T stands for threshold; FDR: false discovery rate. | | | |

| **Table S4. The primer sequences of miRNAs for validation experiments** | | |
| --- | --- | --- |
| Gene_id | Primer | Primer sequence |
| miR-143b-3p | RT | GTCGTATCCAGTGCAGGGTCCGAGGTATTCGCACTGGATACGACGAGCTA |
|  | F | CGGCTGAGATGAAGCACTG |
|  | P | TCGCACTGGATACGACGAGCTA |
| miR-363-3p | RT | GTCGTATCCAGTGCAGGGTCCGAGGTATTCGCACTGGATACGACTACAGAT |
|  | F3 | ACGGAATTGGACGGCATCC |
|  | P | TTCGCACTGGATACGACTACAGATG |
| miR-200a-3p | RT | GTCGTATCCAGTGCAGGGTCCGAGGTATTCGCACTGGATACGACACATCG |
|  | F | ACGCTAACACTGTCTGGTAAC |
|  | P | TTCGCACTGGATACGACACATCG |
| miR-125b-5p | RT | GTCGTATCCAGTGCAGGGTCCGAGGTATTCGCACTGGATACGACTCACAAG |
|  | F | AGCCTCCCTGAGACCCTAA |
|  | P | TTCGCACTGGATACGACTCACAAG |
| miR-140-3p | RT | GTCGTATCCAGTGCAGGGTCCGAGGTATTCGCACTGGATACGACCCGTGG |
|  | F | GCCGTACCACAGGGTAGAA |
|  | P | TCGCACTGGATACGACCCGTGG |
| U6 | qPCR-TYR | GTGCAGGGTCCGAGGT |
|  | U6-RT | AACGCTTCACGAATTTGCGT |
|  | U6-S | CTCGCTTCGGCAGCACA |
|  | U6-A | AACGCTTCACGAATTTGCGT |
| **Abbreviations:** RT, RT primer; F, forward primer; P, probe primer. | | |

| **Table S5a. Top 10 DE miRNAs from HCs vs HDPs** | | | | | | | |
| --- | --- | --- | --- | --- | --- | --- | --- |
| **Gene_id** | **CT (log2)** | **CT (SD)** | **HDP (log2)** | **HDP (SD)** | **Fold Change (log2)** | ***p* value** | **FDR** |
| hsa-miR-129-5p | 3.368 | 0.8 | 0.291 | 0.624 | -3.077 | 3.01E-08 | 1.40E-05 |
| hsa-miR-363-3p | 7.208 | 0.43 | 5.769 | 0.369 | -1.438 | 3.02E-07 | 7.04E-05 |
| hsa-miR-629-5p | 6.732 | 0.5 | 5.269 | 0.503 | -1.463 | 4.01E-06 | 0.000623 |
| hsa-miR-375-3p | 7.056 | 0.7 | 3.781 | 1.337 | -3.274 | 9.11E-06 | 0.000849 |
| hsa-miR-16-5p | 17.219 | 0.43 | 16.172 | 0.33 | -1.047 | 1.15E-05 | 0.000891 |
| hsa-miR-92a-3p | 13.043 | 0.44 | 11.965 | 0.385 | -1.078 | 1.80E-05 | 0.001049 |
| hsa-miR-451a | 11.901 | 0.57 | 10.57 | 0.297 | -1.33 | 1.62E-05 | 0.00108 |
| hsa-miR-151a-5p | 6.649 | 0.48 | 7.7 | 0.322 | 1.052 | 3.44E-05 | 0.001601 |
| hsa-miR-151b | 5.928 | 0.52 | 6.954 | 0.377 | 1.026 | 0.000114 | 0.004429 |
| hsa-miR-484 | 7.453 | 0.72 | 6.083 | 0.557 | -1.37 | 0.000188 | 0.006736 |
| **Abbreviations:** MDPs, methamphetamine-dependent patients; HDPs, heroin-dependent patients; HCs, healthy controls; C stands for Cycle and T stands for threshold; FDR: false discovery rate. | | | | | | | |

| **Table S5b. Top 10 DE miRNAs from HCs vs MDPs** | | | | | | | |
| --- | --- | --- | --- | --- | --- | --- | --- |
| **Gene_id** | **CT (log2)** | **CT (SD)** | **MDP (log2)** | **MDP (SD)** | **Fold Change (log2)** | ***p* value** | **FDR** |
| hsa-miR-451a | 11.901 | 0.57 | 10.143 | 0.688 | -1.757 | 8.62E-06 | 0.0013382 |
| hsa-miR-129-5p | 3.368 | 0.8 | 0.721 | 1.018 | -2.647 | 5.77E-06 | 0.001344 |
| hsa-miR-548ay-5p | 4.452 | 0.35 | 3.109 | 0.519 | -1.343 | 4.67E-06 | 0.0021773 |
| hsa-miR-636 | 2.072 | 0.67 | 0.496 | 0.648 | -1.576 | 4.25E-05 | 0.0028306 |
| hsa-miR-548ae-5p | 4.51 | 0.32 | 3.345 | 0.57 | -1.165 | 5.86E-05 | 0.0030332 |
| hsa-miR-548az-5p | 4.154 | 0.57 | 2.478 | 0.8 | -1.676 | 5.69E-05 | 0.0033169 |
| hsa-miR-548ad-5p | 4.361 | 0.42 | 2.919 | 0.734 | -1.442 | 8.75E-05 | 0.0040798 |
| hsa-miR-16-5p | 17.219 | 0.43 | 16.045 | 0.596 | -1.174 | 0.000108 | 0.0045901 |
| hsa-miR-548ab | 4.018 | 0.53 | 2.638 | 0.699 | -1.38 | 0.000119 | 0.0046109 |
| hsa-miR-548b-5p | 4.15 | 0.47 | 2.814 | 0.708 | -1.336 | 0.000146 | 0.0048538 |
| **Abbreviations:** MDPs, methamphetamine-dependent patients; HCs, healthy controls; C stands for Cycle and T stands for threshold; FDR: false discovery rate. | | | | | | | |

Raw wetstern blotting imaging


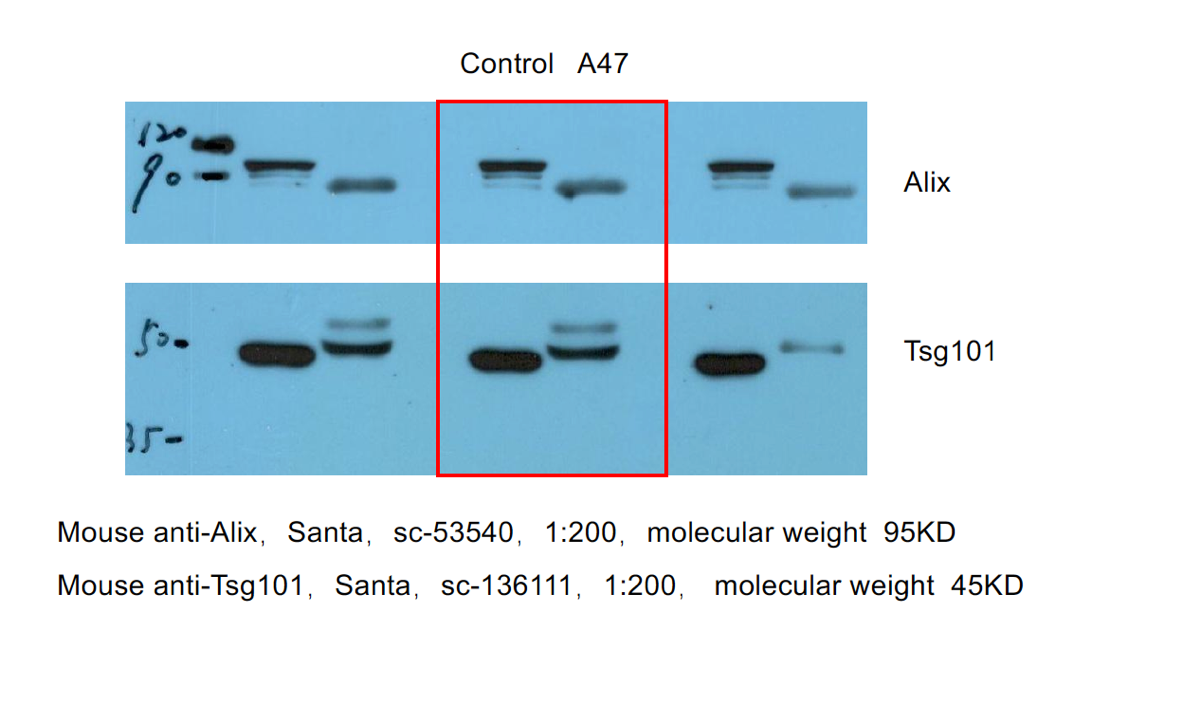

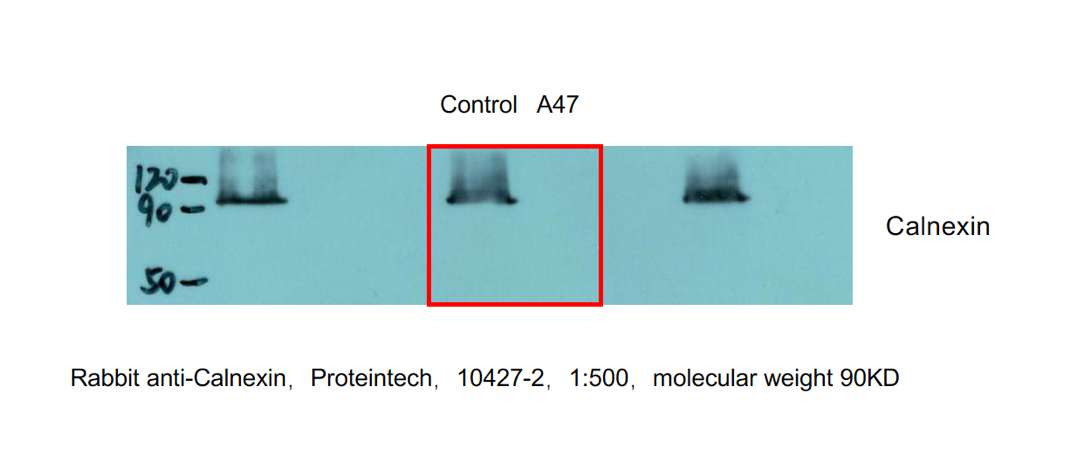

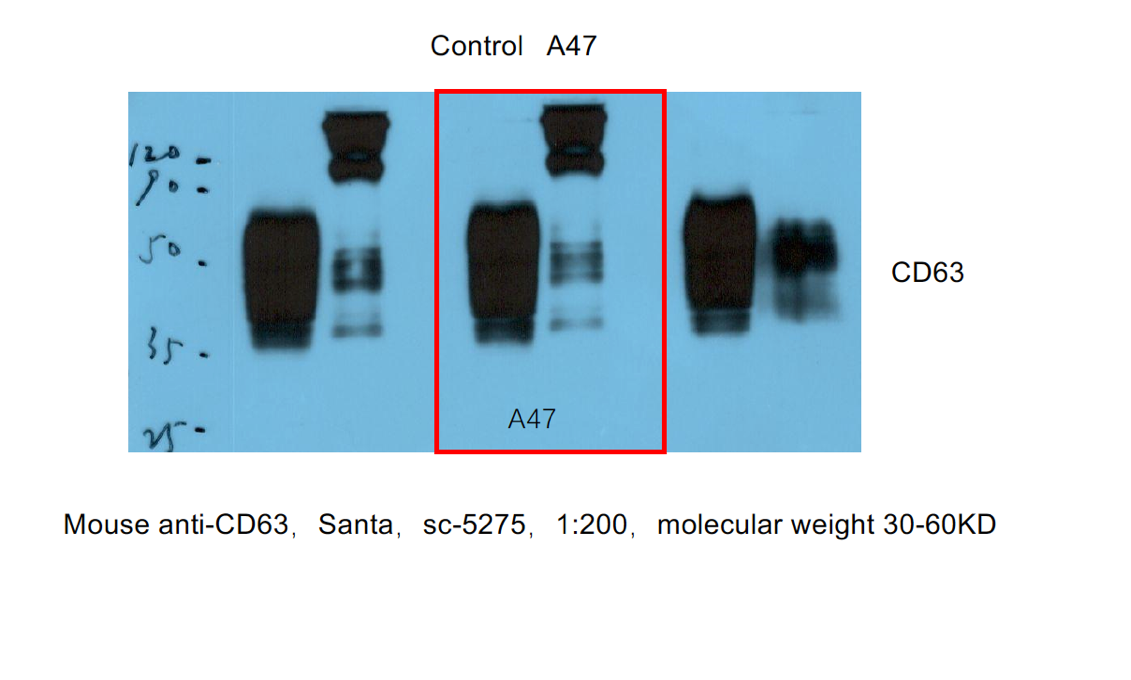

Supplement: Supplementary file 1 — Supplementary Information [file 41598_2021_84501_MOESM1_ESM.docx]
